# HIV-Infected Men Who Have Sex with Men Who Identify Themselves as Belonging to Subcultures Are at Increased Risk for Hepatitis C Infection

**DOI:** 10.1371/journal.pone.0057740

**Published:** 2013-03-04

**Authors:** Amy Matser, Joost Vanhommerig, Maarten F. Schim van der Loeff, Ronald B. Geskus, Henry J. C. de Vries, Jan M. Prins, Maria Prins, Sylvia M. Bruisten

**Affiliations:** 1 Cluster of Infectious Diseases, Public Health Service of Amsterdam, Amsterdam, The Netherlands; 2 Julius Center for Health Science and Primary Health Care, University Medical Center Utrecht, Utrecht, The Netherlands; 3 Department of Medical Microbiology, Academic Medical Center (AMC), Amsterdam, The Netherlands; 4 Center for Infection and Immunology Amsterdam (CINIMA), Academic Medical Center (AMC), Amsterdam, The Netherlands; 5 Department of Clinical Epidemiology, Biostatistics and Bioinformatics, Academic Medical Center, Amsterdam, The Netherlands; 6 Department of Dermatology, Academic Medical Center (AMC), Amsterdam, The Netherlands; 7 Centre for Infectious Disease Control, National Institute of Public Health and the Environment (RIVM), Bilthoven, The Netherlands; 8 Department of Internal Medicine, Division of Infectious Diseases, Academic Medical Center (AMC), Amsterdam, The Netherlands; 9 Department of Experimental Virology, University of Amsterdam, Amsterdam, The Netherlands; Mayo Clinic, United States of America

## Abstract

**Background:**

Hepatitis C virus (HCV) emerged as sexually transmitted infection among HIV-infected men who have sex with men (MSM). We studied whether HCV circulated in identifiable high-risk MSM subcultures and performed phylogenetic analysis.

**Methods:**

HIV-infected MSM were recruited at the sexually transmitted infections (STI) outpatient clinic and a university HIV clinic in Amsterdam, the Netherlands, 2008–2009. Participants completed a detailed questionnaire and were tested for HCV antibodies and RNA, with *NS5B* regions sequenced for analysis of clusters.

**Results:**

Among 786 participants, the median age was 43 (IQR 37–48) years, and 93 (11.8%) were HCV-positive. Seropositivity was associated with belonging to subcultures identified as leather (aOR 2.60; 95% CI 1.56–4.33), rubber/lycra (aOR 2.15; 95% CI 1.10–4.21), or jeans (aOR 2.23; 95% CI 1.41–3.54). The two largest HCV-RNA monophyletic clusters were compared; MSM in cluster I (genotype 1a, n = 13) reported more partners (*P* = 0.037) than MSM in cluster II (genotype 4d, n = 14), but demographics, subculture characteristics and other risk behaviors did not differ significantly between the two clusters.

**Discussion:**

HCV infection is associated with identifiable groups of leather/rubber/lycra/jeans subcultures among HIV-infected MSM. Separate epidemiological HCV transmission networks were not revealed. Active HCV screening and treatment within specific subcultures may reduce HCV spread among all MSM.

## Introduction

During the last decade, hepatitis C virus (HCV) has emerged among HIV-infected men who have sex with men (MSM) in several industrialized countries [1]–[8]. It became clear that, in this population, HCV spreads mainly by sexual transmission [9]. The HCV incidence among HIV-infected MSM rose from 0.9 per 1000 person-years in 1990 to 23.4 per 1000 person-years in 2007 [10].

Epidemiological studies showed that HCV infection in HIV-infected MSM is associated with unprotected anal intercourse, multiple sexual partners, recreational non-injecting drug use, and rough sexual techniques (e.g., sharing of sex toys and brachioproctic insertion, also known as fisting) [1], [11], [12], but these studies did not link these factors to any particular MSM subculture.

Although HCV is transmitted through a large international network, isolates tend to cluster by country or region [13]. Among MSM in Amsterdam, the predominant HCV genotypes are 1a and 4d [3], and the strain of genotype 4 found in MSM is phylogenetically distinct from the strain found in injecting drug users or migrants from Egypt [14]. These findings led us to question whether, within a country or a city, HCV strains would cluster according to MSM subpopulations that arise by clustering of MSM who share the same characteristics. One of the possible ways MSM cluster together is according to lifestyle or subculture, e.g., MSM who belong to the leather scene might cluster together. Literature about the existence of subpopulations is limited. To our knowledge, only the leather scene has been described [31]. Based on the themes of gay bars, clubs and parties, it can be deduced that a number of subpopulations or subcultures exist in the gay community. By searching the internet and the agendas of venues in Amsterdam we identified several possible subpopulations or subcultures. Besides the leather scene, we identified a subpopulation of MSM who wear military or other uniforms, a group who wears rubber/lycra clothing and have their own websites and parties, and a group of MSM wearing jeans who also have their own parties and partially overlap with the leather scene. We also identified a sports community of younger MSM who wear sports outfits and also have their own parties and dress code. We hypothesised that assortative mixing, or partnership formation within subgroups, is common and that this mixing pattern might result in HCV being more prevalent in certain subpopulations than in others.

Especially in the current epidemic phase, insight into the transmission network can contribute to more effective screening and treatment in the near future. Screening and treatment can, in turn, have a major impact on the prevalence by reducing transmission [15]. In the Netherlands, HCV screening currently occurs at STI clinics and at the HIV treatment centres. In addition to current practice, the initiation of outreach programs and on location screening might contribute to further identification of HCV-infected MSM. To determine what the most important target groups are for these types of interventions, it is important to determine characteristics that help identify subgroups. The objective of this study was to find such subgroups, identifiable by lifestyle or subculture. Furthermore, we performed phylogenetic analysis to examine whether the presence of certain specific HCV strains was associated with specific MSM subpopulations.

## Methods

### Setting & Participants

This study is part of a larger cross-sectional study focussing on spread of sexually transmitted infections (STI) via sexual networks; its aims, population, and methods are discussed elsewhere [16]. The study population was recruited from MSM attending the STI outpatient clinic of the Public Health Service of Amsterdam and the HIV outpatient clinic of the Academic Medical Center (AMC), Amsterdam, the Netherlands. MSM were defined as men who reported any sexual contact with men during the six months preceding the clinic visit. They were eligible for participation if they were at least 18 years old, could understand written Dutch or English, and provided written informed consent.

Those recruited at the STI outpatient clinic included MSM with and without STI symptoms. They could participate more than once if they revisited the clinic because of a possibly new STI episode. The recruitment period was from July 2008 to August 2009 and was briefly interrupted twice by logistical conflicts with another study.

The participants from the HIV outpatient clinic were recruited from a cohort of MSM visiting for routine 3- or 6-monthly clinic visits. They were included based on the same criteria as used in the STI clinic, but none of them had STI symptoms, having served previously (October 2007 through August 2008) in a study investigating STI prevalence in asymptomatic visitors of the HIV clinic [17].

At both locations, participants were screened for *Chlamydia trachomatis*, *Neisseria gonorrhoeae*, and *Treponema pallidum*, and when appropriate, they were screened for Hepatitis B and HIV, according to the standard procedures of the STI outpatient clinic. At the STI clinic, all HIV-infected MSM or MSM who opted-out for the HIV test were tested for the presence of HCV, unless the HCV seropositive status was known from previous testing at the clinic. At the HIV clinic, HCV antibody testing (AxSYM® HCV 3.0, Abbott Laboratories, Abbott Park, Illinois, USA) was done at the first visit and subsequently, when suspicious for HCV infection e.g. when serum alanine aminotransferase (ALT) levels were elevated. The current analysis was restricted to the first visit of HIV-infected participants. The study was approved by the medical ethics committee of the AMC.

### HCV Testing

Serum samples were stored at −20°C and tested for HCV antibodies with a third-generation commercial microparticle enzyme immunoassay (MEIA), AxSYM® HCV 3.0 (Abbott Laboratories, Abbott Park, Illinois, USA). When positive for HCV antibodies, sera were qualitatively tested for HCV RNA using an in-house real-time PCR assay targeting the highly conserved 5′ untranslated region (UTR) of the HCV genome [18]. RNA was extracted from 200 µl serum using the TriPure method (Roche Diagnostics, Almere, The Netherlands) and eluted in a volume of 50 µl. Real-time PCR mixes (25 µl total volume) contained 12.5 µl of 2× Reaction Mix (Superscript One-Step RT PCR kit, Invitrogen, USA), 0.2 µl of forward and reverse primers, 100ng/µl [19], and 0.1 µl of FAM-labelled HCV TaqMan® probe, 100 ng/µl [18]. Real-time runs were performed on a Rotor Gene (Qiagen, Germany) using the following cycling conditions: 15 min at 45°C, 2 min at 95°C, followed by 40 cycles of 15 sec at 95°C, 30 sec at 50°C, and 30 sec at 60°C. Samples with a threshold cycle (Ct) ≤37 and an expected S-curve were considered positive. Samples with Ct between 37 and 40 were retested and considered positive when Ct ≤40.

### Sequence Analysis

After detection of HCV RNA, viral genotype was determined using nested PCR targeting HCV core [20] and *NS5B* [18] regions. We obtained *NS5B* sequences using an ABI3130 Genetic Analyzer (Applied Biosystems, Foster City, California, USA) and created alignments with GenBank reference sequences using Mega v5.0 (GenBank Accession Nos. JQ917721-JQ917762) [21]. A phylogenetic tree was constructed by the neighbour-joining method, using the Tamura-Nei substitution model [22] with γ-distribution (α = 0.40). Inferred phylogenies were tested with 1000 bootstrap replications.

### Questionnaire

Participants completed a computer-assisted self-interview. The questionnaire reflected characteristics and behavior in the six months preceding the recruitment visit. It addressed demographics and sexual behaviors in up to four specified partnerships: one self-defined steady partner and the most recent three other partners. These others could be self-defined as steady, known (i.e., traceable), or anonymous (i.e., non-traceable). In the questionnaire, lifestyle was determined by asking whether the participant characterized himself by code of dressing or as belonging to a certain social stream or subculture within the gay community. Based on a knowledge from the internet and the agendas of bars, clubs and parties we provided the following options: casual, formal, alternative, drag, leather, military, sports, trendy, punk/skinhead, rubber/lycra, gothic, bear, jeans, skater and other if none of these characteristics applied. In the last case, MSM had the opportunity to give their own description. No a priori definition of lifestyle was given to allow participants to subjectively determine what subculture most applied to them. In the current study we only used subcultures that are typical subcultures in the MSM community and to which specific meeting venues or parties were linked. These included leather, rubber/lycra, military, jeans, and sports subcultures. Multiple answers were possible. Questions about sexual risk behaviors were asked about each of the specified partnerships.

### Statistical Analysis

To examine whether the presence of HCV antibodies was associated with characteristics that could easily identify the subpopulation(s) most at risk for HCV we performed initial data analysis, including χ^2^-tests for independence for dichotomous and categorical variables and Mann-Whitney *U* tests for continuous variables. Fisher’s exact tests were performed when the expected value in a cell was less than one. Furthermore, we performed univariable and multivariable logistic regression analysis. In advance, we selected a set of variables for the analyses that could help identifying individuals at risk without asking questions. Multivariable analysis was performed by including all selected variables into the model and by using backward stepwise regression. The continuous variable age was modelled as restricted cubic splines with knots at the 2.5^th^, 25^th^, 50^th^, 75^th^, and 97.5^th^ percentiles [23]. To examine whether specific sexual risk behavior was associated with the high-HCV risk subpopulation(s), we performed multivariable logistic regression analysis without a backward selection algorithm, with high-risk subcultures as outcome and various sexual risk behaviors as covariates. The results of this analysis are shown in Appendix I.

Phylogenetic analysis was performed to identify monophyletic clusters (bootstrap>70%) of more than 10 individuals. The characteristics and also risk behavior within the resulting clusters were analysed and compared with each other and with the remainder, a group consisting of a smaller cluster and singletons. We used χ^2^-tests and Fisher’s exact tests for dichotomous and categorical variables, and Kruskal-Wallis tests for continuous variables. Analysis was done using STATA 11.1 (STATA Corp., College Station, TX, USA) and R version 2.14.2 [24].

## Results

### Study Population

Of the 2694 recruited MSM, 788 individuals (29.3%) were HIV-infected. Of these, two were excluded because their questionnaire data were incomplete, resulting in a study population of 786 HIV-infected MSM. Of these, 586 were recruited at the STI outpatient clinic and 200 at the HIV outpatient clinic. The median age of the total study population was 43 (IQR 37–48), and 71.3% of the population was Dutch (Table 1). MSM recruited at the STI clinic were younger, less often Dutch, reported higher sexual risk behavior and were more often diagnosed with STI than MSM recruited at the HIV clinic (Table 1). Sexual risk behavior in this HIV-infected population was high, with receptive unprotected anal intercourse (UAI) reported by 51.3%, receptive fisting by 14.5%, group sex by 38.7%, and recreational drugs, excluding poppers, by 42.3% (Table 1). Two individuals reported a history of injecting drug use; one was HCV-antibody-positive (1.1% of all HCV-seropositive participants) and one was HCV-antibody-negative (0.1% of all HCV-seronegative participants). Characteristics of the population according to HCV serostatus are shown in table 2.

**Table 1:** Characteristics of 786 HIV-infected MSM, by recruitment location, who visited the STI outpatient clinic of the Public Health Service or the HIV outpatient clinic of the Academic Medical Center in Amsterdam, the Netherlands, 2008–2009.

		Total population(N = 786)	GGD(N = 586)	AMC(N = 200)	p
**Demographics**					
Median age in years (IQR)		43 (37–48)	41 (36–47)	47 (42–53)	<0.001
Ethnicity					<0.001
	Dutch	560 (71.3%)	394 (67.2%)	166 (83.0%)	
	Western, non-Dutch	98 (12.5%)	84 (14.3%)	14 (7.0%)	
	Non-western	128 (16.3%)	108 (18.4%)	20 (10.0%)	
**Subculture**					
Casual		693 (88.2%)	517 (88.2%)	176 (88.0%)	0.932
Leather		153 (19.5%)	123 (21.0%)	30 (15.0%)	0.065
Military		71 (9.0%)	60 (10.2%)	11 (5.5%)	0.044
Sport		171 (21.8%)	143 (24.4%)	28 (14.0%)	0.002
Rubber/lycra		57 (7.3%)	45 (7.7%)	12 (6.0%)	0.429
Jeans		251 (31.9%)	53 (26.5%)	198 (33.8%)	0.056
**Sexual behaviour**					
Median no. of partners in the preceding 6 months (IQR)		8 (3–20)	10 (5–25)	3 (1–8)	<0.001
Receptive UAI^1^		400 (51.3%)	58 (29.9%)	342 (58.4%)	<0.001
Insertive UAI^1^		348 (44.6%)	303 (51.7%)	45 (23.2%)	<0.001
Receptive fisting		113 (14.5%)	100 (17.2%)	13 (6.7%)	<0.001
Insertive fisting		126 (16.2%)	109 (18.7%)	17 (8.8%)	0.001
Group sex		301 (38.7%)	252 (43.2%)	49 (25.3%)	<0.001
Poppers use		416 (53.5%)	347 (59.5%)	69 (35.6%)	<0.001
Other drug use^2^		329 (42.3%)	289 (49.6%)	40 (20.6%)	<0.001
**Sexually transmitted infections diagnosis**					
Syphilis^3^		377 (48.0%)	320 (54.6%)	57 (28.5%)	<0.001
Chlamydia		131 (16.7%)	115 (19.6%)	16 (8.1%)	<0.001
Gonorrhea		106 (13.5%)	104 (17.8%)	2 (1.0%)	<0.001
HCV		93 (11.8%)	81 (13.8%)	12 (6.0%)	0.003

NOTE: Numbers do not always add up to the column totals due to missing data; there was 1 missing value for the age variable, 6 missing values for receptive and insertive UAI, 9 missing for other risk behavior variables, and 2 missing for the chlamydia and gonorrhoea variables.

NOTE: The subculture characteristics are not mutually exclusive.

HIV = human immunodeficiency virus; STI = sexually transmitted infection; IQR = interquartile range; UAI = unprotected anal intercourse.

1 P values were calculated for recruitment at the STI clinic versus the HIV clinic and considered significant when *p*<0.05. ^2^ Recreational use of cocaine, XTC, gamma hydroxybutyrate (GHB), ketamines, amphetamines, or methylamphetamines before or during sexual contact. ^3^ Based on serological evidence.

**Table 2:** Characteristics of 786 HIV-infected men who have sex with men, by hepatitis C antibody status, who visited the STI outpatient clinic of the Public Health Service or the HIV outpatient clinic of the Academic Medical Center in Amsterdam, 2008–2009.

		HCV-antibody-positive (%)N = 93 (11.8%)	HCV-negative (%)N = 693 (88.2%)	P^1^
Recruitment location				0.003
	STI clinic	81 (87.1%)	505 (72.9%)	
	HIV clinic	12 (12.9%)	188 (27.1%)	
**Demographics**				
Median age in years (IQR)		44 (39–49)	42 (37–48)	0.062
Ethnicity				0.092
	Dutch	71 (76.3%)	489 (70.6%)	
	Western, non-Dutch	14 (15.1%)	84 (12.1%)	
	Non-western	8 (8.6%)	120 (17.3%)	
**Subculture**				
Casual		79 (85.0%)	614 (88.6%)	0.306
Leather		40 (43.0%)	113 (16.3%)	<0.001
Military		16 (17.2%)	55 (7.9%)	0.003
Sport		32 (34.4%)	139 (20.1%)	0.002
Rubber/lycra		18 (19.4%)	39 (5.6%)	<0.001
Jeans		50 (53.8%)	201 (29.0%)	<0.001
**Sexual behavior**				
Median no. of partners in the preceding 6 months (IQR)		10 (5–30)	8 (3–20)	0.001
Receptive UAI		67 (72.0%)	333 (48.5%)	<0.001
Insertive UAI		60 (64.5%)	288 (41.9%)	<0.001
Receptive fisting		27 (29.0%)	86 (12.6%)	<0.001
Insertive fisting		27 (29.0%)	99 (14.5%)	<0.001
Group sex		59 (63.4%)	242 (35.4%)	<0.001
Poppers use		63 (67.7%)	353 (51.6%)	0.003
Other drug use 2		61 (65.6%)	268 (39.2%)	<0.001
**Sexually transmitted infections diagnosis**				
Syphilis 3		64 (68.8%)	313 (45.2%)	<0.001
Chlamydia		18 (19.4%)	113 (16.4%)	0.466
Gonorrhea		14 (15.1%)	92 (13.3%)	0.645

NOTE: Percentages do not always add up to 100% due to rounding.

NOTE: Numbers do not always add up to the column totals due to missing data; there was 1 missing value for the age variable, 6 missing values for receptive and insertive UAI, 9 missing for other risk behavior variables, and 2 missing for the chlamydia and gonorrhoea variables.

NOTE: The subculture characteristics are not mutually exclusive.

HIV = human immunodeficiency virus; STI = sexually transmitted infection; IQR = interquartile range; UAI = unprotected anal intercourse.

1 P values were calculated for HCV antibody positives versus HCV antibody negatives and considered significant when <0.05.

2 Recreational use of cocaine, XTC, gamma hydroxybutyrate (GHB), ketamines, amphetamines, or methylamphetamines before or during sexual contact.

3 Based on serological evidence.

### Determinants of HCV Seropositive Status

HCV antibodies were present in 93 of 786 HIV-infected MSM; the HCV prevalence was 11.8% (95% CI 9.6–14.1%). We analyzed whether easily identifiable characteristics (i.e., age, ethnicity, lifestyle/subculture variables) were associated with HCV seropositivity. In univariable analysis, age and ethnicity were not significantly associated, whereas several subculture variables were (Table 3). In multivariable analysis, men self-typed as leather (adjusted odds ratio [aOR] 2.60; 95% CI 1.56–4.33), rubber/lycra (aOR 2.15; 95% CI 1.10–4.21), or jeans (aOR 2.23; 95% CI 1.41–3.54) were more likely to be HCV-seropositive than men who did not identify with those subcultures (Table 3). Interactions between the three variables were tested, but did not improve the model (*P* = 0.330). Among 786 HIV-infected persons, 328 (41.7%) MSM belonged to one of the high-risk subcultures (leather, rubber/lycra or jeans); among HCV-negatives, 261 (37.7%) of the 693 and among HCV-infected MSM, 67 (72.0%) of the 93 belonged to a high-risk subculture. The likelihood of being HCV seropositive increased when MSM belonged to multiple subcultures. Compared to not belonging to a subculture, the OR was 1.73 (95% CI 0.91–3.27) when MSM belonged to one subculture and increased to 4.56 (95% CI 2.59–8.04) when they belonged to two subcultures, and to 6.70 (95% CI 3.36–13.34) for when they belonged to three or more subcultures. Eighty percent of the MSM who belonged to one of the identified subcultures themselves reported at least one partner who also belonged to one of the subcultures, while only 26.6% of the MSM who did not belong to any of the subcultures reported a partner who belongs to a subculture.

**Table 3:** Identifiable determinants of hepatitis C seropositive status among 786 HIV-infected men who have sex with men, of whom 93 were hepatitis C seropositive, in Amsterdam, 2008–2009.

		OR (95% CI) 1	p	aOR (95%CI) 2	p	aOR (95%CI) 3	p
**Demographics**							
Age in years 4			0.082		0.310		
	35	0.78 (0.48–1.27)		0.86 (0.53–1.42)			
	40	1.00		1.00			
	45	1.26 (0.90–1.75)		1.26 (0.79–2.01)			
	50	1.50 (0.86–2.60)		1.25 (0.61–2.57)			
Ethnicity			0.100		0.310		
	Dutch	1.00		1.00			
	Western, non-Dutch	1.15 (0.62–2.13)		1.13 (0.59–2.16)			
	Non-western	0.46 (0.22–0.98)		0.56 (0.25–1.25)			
**Subculture**							
Leather		3.87 (2.45–6.12)	<0.001	2.59 (1.50–4.47)	<0.001	2.60 (1.56–4.33)	<0.001
Military		2.41 (1.32–4.41)	0.004	0.74 (0.36–1.53)	0.420		
Sport		2.09 (1.31–3.33)	0.002	1.77 (1.03–3.03)	0.037		
Rubber/lycra		4.02 (2.19–7.39)	<0.001	2.03 (1.01–4.08)	0.046	2.15 (1.10–4.21)	0.026
Jeans		2.85 (1.83–4.42)	<0.001	1.95 (1.20–3.17)	0.007	2.23 (1.41–3.54)	<0.001

NOTE: There was 1 missing value in the age variable.

HIV = human immunodeficiency virus; OR = odds ratio; CI = confidence interval; aOR = adjusted odds ratio.

1 Odds ratio resulting from univariable analysis.

2 Odds ratio adjusted for all variables.

3 Odds ratio adjusted for variables in the model after backward selection.

4 Modelled as restricted cubic spline with knots at the 2.5^th^, 25^th^, 50^th^, 75^th^, and 97.5^th^ percentiles.

In the total group of 786 MSM, we examined whether the high-risk subcultures were associated with particular sexual behaviors. In multivariable analysis, they were more often linked with a higher number of partners (P<0.001), receptive fisting (aOR 2.82; 95% CI 1.59–5.02), and use of recreational drugs (aOR 1.48; 95% CI 1.03–2.12) (Table 4).

**Table 4:** Sexual behavior associated with a high-HCV-risk subculture (i.e., leather, rubber/lycra, or jeans) among 786 HIV-infected MSM, Amsterdam, 2008–2009.

		High-HCV-risk subculture(N = 328)	Other subculture(N = 458)	OR^1^	(95% CI)	p	aOR^2^	(95%CI)	p
No. of partners in the preceding 6 months^3^		10 (IQR 4–30)	6 (IQR 3–15)			<0.001			<0.001
	1			1.00			1.00		
	5			0.93	(0.73–1.18)		0.84	(0.60–1.17)	
	10			1.72	(1.16–2.56)		1.35	(0.87–2.10)	
	25			2.43	(1.65–3.57)		1.80	(1.16–2.79)	
Receptive UAI^4^		186/400 (46.5%)	139/380 (36.6%)	1.51	(1.13–2.01)	0.005	0.97	(0.66–1.42)	0.860
Insertive UAI^4^		161/348 (46.3%)	164/432 (38. 0%)	1.41	(1.06–1.87)	0.020	0.88	(0.61–1.29)	0.520
Receptive fisting		79/113 (69.9%)	245/664 (36.9%)	3.97	(2.58–6.12)	<0.001	2.82	(1.59–5.02)	<0.001
Insertive fisting		77/126 (61.1%)	247/651 (37.9%)	2.57	(1.74–3.80)	<0.001	1.08	(0.63–1.85)	0.790
Group sex		158/301 (52.5%)	166/476 (34.9%)	2.06	(1.53–2.77)	<0.001	1.11	(0.77–1.61)	0.570
Poppers use		199/416 (47.8%)	125/361 (34.6%)	1.73	(1.30–2.31)	<0.001	1.22	(0.87–1.70)	0.240
Drug use^5^		172/329 (52.3%)	152/448 (33.9%)	2.13	(1.59–2.86)	<0.001	1.48	(1.03–2.12)	0.032

NOTE: Numbers do not always add up to the column totals due to missing data; there were 6 missing values in receptive and insertive UAI and 9 missing in variables for fisting, group sex, poppers, and drug use.

1 Odds ratio.

2 adjusted odds ratio.

3 Modelled as restricted cubic spline, and thus no group size or OR could be reported; instead the median and IQR and p-values for the logistic regression are provided.

4 Unprotected anal intercourse.

5 Recreational use of cocaine, XTC, gamma hydroxybutyrate (GHB), ketamines, amphetamines, or methylamphetamines before or during sexual contact.

### Phylogenetic Analysis

**Figure 1:**
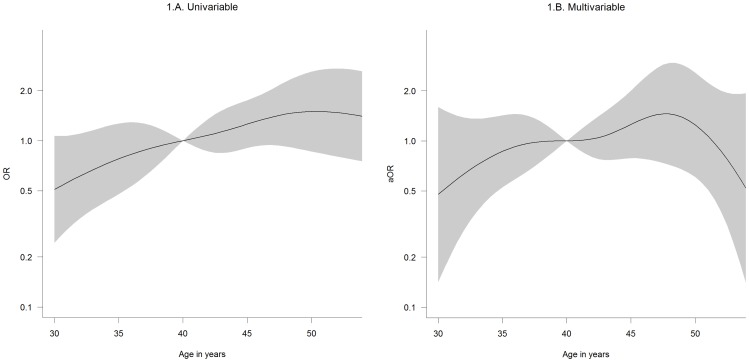
Phylogenetic tree of 42 HCV *NS5B* sequences obtained from HIV-infected MSM in Amsterdam. Three clusters were identified: cluster I with genotype 1a (n = 13), cluster II with genotype 4d (n = 14), a smaller cluster III with genotype 1a (n = 7), and 8 singletons. Self-identified subcultures are indicated as follows: leather in black; jeans in yellow; rubber/lycra in green, sports in red; no subculture in white. History of injecting drug use is indicated by a needle. More than one subculture per person is possible.

Serum samples from the 93 HCV-seropositive MSM were tested for the presence of HCV RNA, and HCV RNA was detected in 46 (49.5%). The RNA-positive and RNA-negative men did not differ as to demographics or STI coinfections. *NS5B* sequences were obtained from 42 (91.3%) of the 46 RNA-positive samples, yielding HCV genotypes 1a (57.1%), 1b (7.1%), 3a (2.4%), and 4d (33.3%). Phylogenetic analysis revealed two monophyletic clusters of n = 13 and n = 14 (clusters I and II, respectively), one smaller cluster of n = 7 (cluster III) and 8 singletons (Figure 1).

Descriptive analysis was performed on clusters I and II and on the remainder group of 15. The men in the three groups did not differ significantly by age or ethnicity (Table 5). Spread of the different HCV strains was not restricted to the specific subcultures (i.e., leather, military, rubber/lycra, or jeans), as there was no significant association between subcultures and HCV cluster. Those in the remainder group were more often sports-type MSM than men in clusters I or II: 60.0% vs. 15.4% and 21.4% (*P* = 0.023) (Table 5). A significant difference between cluster I and II was seen for the median number of partners in the preceding six months (*P* = 0.037): in cluster I, it was 25 (IQR 15–50) and in cluster II, 5.5 (IQR 1–25); in the remainder group, it was 8 (IQR 5–30). In cluster analysis that included the remainder, this difference among groups had only borderline significance (*P* = 0.059). Insertive UAI was more often reported by men in the remainder group: 93.3% vs. 61.5% in cluster I and 35.7% in cluster II (*P* = 0.005), but the difference between cluster I and II was not significant (*P* = 0.180). Other sexual risk behavior (i.e., receptive UAI, fisting, group sex, use of poppers and other drugs), a history of syphilis infection, and the occurrence of chlamydia and gonorrhea did not differ significantly between the two monophyletic clusters and the remainder group.

**Table 5:** Epidemiological characteristics of 42 men who have sex with men who tested hepatitis C RNA-positive, by phylogenetic cluster, Amsterdam, 2008–2009.

		Cluster I(N = 13)	Cluster II(N = 14)	Other(N = 15)	*P* 1 *	*P* 2†
**Demographics**						
Recruitment location					0.077	0.149
	STI clinic	13 (100.0%)	11 (78.6%)	14 (93.3%)		
	HIV clinic	0	3 (21.4%)	1 (6.7%)		
Median age in years (IQR)		47 (40–49)	48 (44–52)	44 (39–46)	0.436	0.178
Ethnicity					0.995	0.954
	Dutch	10 (76.9%)	11 (78.6%)	10 (66.7%)		
	Western, non-Dutch	2 (15.4%)	2 (14.3%)	3 (20.0%)		
	Non-western	1 (7.7%)	1 (7.1%)	2 (13.3%)		
**Subculture**						
Leather		5 (38.5%)	8 (57.1%)	7 (46. 7%)	0.332	0.621
Military		1 (7.7%)	4 (28.6%)	5 (35.7%)	0.163	0.248
Sport		2 (15.4%)	3 (21.4%)	9 (60.0%)	0.686	0.023
Rubber/lycra		3 (23.1%)	5 (35.7%)	1 (6.7%)	0.472	0.160
Jeans		6 (46.2%)	8 (57.1%)	8 (53.3%)	0.568	0.846
**Sexual behavior**						
Median no. of partners in the preceding 6 months (IQR)		25 (15–50)	5.5 (1–25)	8 (5–30)	0.037	0.059
Receptive UAI		11 (84.6%)	10 (71.4%)	10 (66.7%)	0.410	0.543
Insertive UAI		8 (61.5%)	5 (35.7%)	14 (93.3%)	0.180	0.005
Receptive fisting		2 (15.4%)	6 (42.9%)	3 (20.0%)	0.118	0.213
Insertive fisting		3 (23.1%)	5 (35.7%)	4 (26.7%)	0.472	0.752
Group sex		8 (61.5%)	8 (57.1%)	10 (66.7%)	0.816	0.870
Poppers use		9 (69.2%)	10 (71.4%)	9 (60.0%)	0.901	0. 786
Drug use 3		7 (53.9%)	7 (50.0%)	12 (80.0%)	0.842	0.194
**Sexually transmitted infections diagnosis**						
Syphilis 4		10 (76.9%)	11 (78.6%)	9 (60.0%)	0.918	0.472
Chlamydia		3 (23.1%)	1 (7.1%)	4 (26.7%)	0.244	0.370
Gonorrhea		1 (7.7%)	3 (21.4%)	3 (20.0%)	0.315	0.576

NOTE: The subculture characteristics are not mutually exclusive.

RNA = ribonucleic acid; STI = sexually transmitted infection; HIV = human immunodeficiency virus; IQR = interquartile range; UAI = unprotected anal intercourse;

1 *P*-value for χ^2^-tests and Kruskal-Wallis tests of cluster I and II;

2 *P*-value for χ^2^-tests and Kruskal-Wallis tests of cluster I, II, and the remainder group;

3 Recreational use of cocaine, XTC, gamma hydroxybutyrate (GHB), ketamines, amphetamines, or methylamphetamines before or during sexual contact;

4 Based on serological evidence.

## Discussion

In this study of 786 HIV-infected MSM, we showed that MSM belonging to the leather, rubber/lycra, and jeans subcultures were more likely to be HCV-seropositive than MSM who did not pursue those lifestyles. Moreover, high-risk sexual behavior was more common among these MSM than among those not belonging to these subcultures. It was remarkable that 72.0% of the men who were HCV-seropositive belonged to one of the high-risk subcultures, while only 37.7% of the HCV-negative HIV-infected population belonged to one of these subpopulations. The analysis of monophyletic clusters of men who were HCV RNA-positive did not show separate networks for HCV transmission. HCV genotypes in the remainder group were found to be associated with the sports scene, but because the remainder was a composite of a smaller cluster and singletons, this finding did not represent a transmission network. Our results did not confirm the hypothesis that, within a city, HCV strains from MSM with the same lifestyle would cluster together.

A strength of this study was the large amount of available epidemiological data. Participants completed a detailed questionnaire that was designed to study sexual networks. We therefore had the opportunity to study determinants of HCV that are useful for subgroup identification. No a priori definition of lifestyle was given to allow participants to subjectively determine what subculture most applied to them. Therefore, MSM who felt they belonged to a subculture because they visited venues associated with it or met partners who belonged to the subculture, but for example, did not use the subcultures dress code, could still be identified as being part of the subculture. There was a large agreement between self-defined subculture and subculture of the partners, supporting the robustness of the definition of subculture.

The presence of HCV RNA was tested with a real-time PCR targeting the highly conserved 5′UTR of the HCV genome, which has a detection limit similar to the nested *NS5B* PCR that was used to generate sequences. HCV-seropositive MSM who were RNA-negative were likely to have cleared the infection, either spontaneously or by antiviral therapy, although the spontaneous clearance rate among HIV-infected individuals is low [25]. The genotype distribution found in this study was in accordance with earlier studies that included HCV-infected MSM in Amsterdam [11], [26], We may have missed MSM with acute HCV infection, because HCV status was determined by HCV antibody screening, and RNA testing was performed only in those who were antibody-positive. A delay in formation of HCV antibodies after HCV infection has been described previously in HIV-coinfected men by Thomson *et al*. [27]. In a bi-annual anonymous survey held at the STI clinic in Amsterdam, prevalence of acute HCV infections (antibody negative, RNA positive) was 1.8% among HIV+ MSM in the period 2008–2009 [Unpublished data]. A second and probably more important limitation was that the monophyletic clusters were relatively small. We therefore performed univariable analysis only on the two largest clusters and a remainder group consisting of a smaller cluster and singletons. Another concern is that MSM enrollment at two different facilities yielded slightly divergent groups. Those recruited at the STI clinic had all been previously involved in sexual risk behavior, as indicated by their interest in STI care. However, those recruited the HIV clinic had not necessarily engaged in risk behavior; in fact, such behavior was much lower among them. Because most participants were recruited at the STI clinic, the study population is a non-random sample of the general HIV-infected population. We do not know whether the associations between subcultures and HCV seropositivity found in the study population are similar to the associations in the general population. The reported effects of subcultures on HCV seropositivity may deviate (ie, under- or overestimation) from the true effects in the general HIV-infected population.

Finally, questionnaires reflected sexual risk behavior in the six months preceding participation. As HCV infection may have occurred before that, responses about risk behavior may not reflect behavior at actual time of infection. This time lag could also apply to responses about identification with lifestyle or subculture, although such identification tends to be more stable over time.

Our results are in line with several previous studies showing that multiple sexual partners, receptive unprotected anal intercourse, group sex, rough sexual techniques (e.g., fisting), and recreational drug use were associated with HCV infection [1], [11], [12]. We showed that these factors were likewise associated with the high-risk subcultures of leather, rubber/lycra and jeans-type MSM. Nevertheless, such behaviors were not the main interest of this study, because their role in subculture identification is more complicated than the role of demographic or lifestyle factors.

Little is known about subcultures within the MSM population. A few studies have described specifically the leather scene [28]–[31], but there is no literature on the other subcultures. The leather scene is a self-defined lifestyle characterized by leather clothing, rough sexual activities, heightened valuation of hypersexuality, and adherence to sexual control dynamics (e.g., dominance and submissiveness) [28]–[30], in which unprotected anal intercourse is common [31]. The fact that mixing of subpopulations occurs is supported by the notion that many MSM reported to belong to more than one subculture and HCV spread was not restricted to just one subculture.

Due to the high risk behavior in the identified subcultures, it is possible that HCV was introduced in the MSM population by one or more of these subcultures. To test this hypothesis, temporal data of several decades on MSM subcultures is needed. To our knowledge, this data is not available. A molecular clock analysis was therefore not performed, given that the time interval between the emergence of the leather subculture and the recent HCV epidemic is relatively short, and also the sample size was limited. More extensive research is needed into the behavioral characteristics of subcultures and the extent to which subcultures mix. More information may lead to an improved understanding of risk behavior and the accompanying spread of HCV and also of HIV and other STI within and between subpopulations. We suggest that among MSM who are sexually active within such subgroups HCV screening should be intensified. In addition to the current screening practices at STI clinics and HIV treatment centres, on location screening initiatives, in specific places where such subgroups meet, could be an efficient way to achieve this goal.

Currently, the HCV outbreak is an epidemic mainly in a restricted subpopulation, but it is unknown how this epidemic will evolve. In this study we used HCV seroprevalent cases. It would be interesting for future studies to examine whether HCV reinfection, which is common among HIV-infected MSM, more often occurs in the high risk populations. While it is assumed that sexually transmitted HCV spreads only in HIV-infected MSM, there have been a few case reports of HCV infection in the absence of HIV [32]. In Amsterdam, the prevalence of HCV among HIV-infected MSM seems to be levelling off since 2011 [33], suggesting that the incidence is declining, however, the incidence of reinfection among successfully treated MSM is very high (15.2 per 100 person years) [26], demonstrating that HCV transmission occurs.

To conclude, we found that HIV-infected MSM belonging to the leather, rubber/lycra and jeans subcultures are at increased risk of acquiring HCV, compared to MSM who do not belong to these subcultures. For public health purposes, we provide a clear description of the MSM population in which most HCV infections might be detected. Implementation of active screening, followed by treatment, in the leather, rubber/lycra and jeans scenes, may reduce HCV incidence and prevalence and the future HCV disease burden among MSM.
