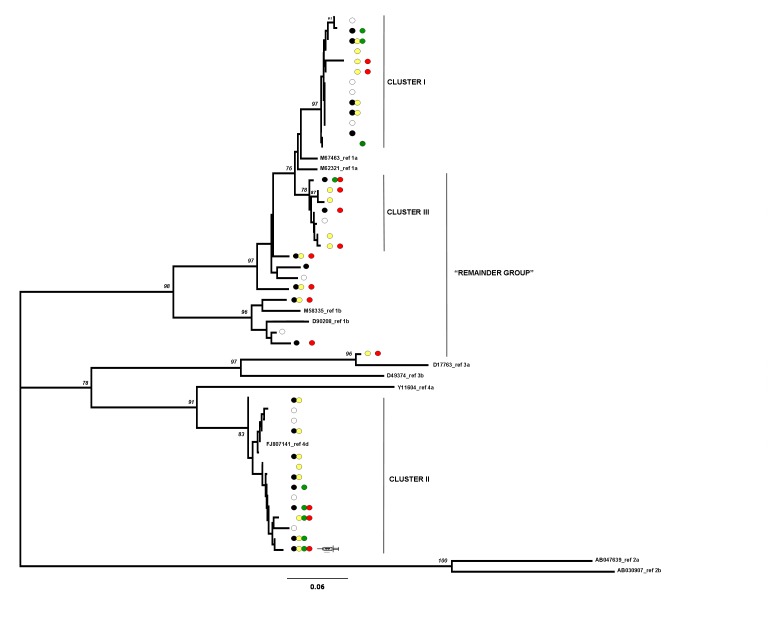# Correction: HIV-Infected Men Who Have Sex with Men Who Identify Themselves as Belonging to Subcultures Are at Increased Risk for Hepatitis C Infection

**DOI:** 10.1371/annotation/68280773-67d3-4d2a-8921-34ca2ba8b41f

**Published:** 2013-04-11

**Authors:** Amy Matser, Joost Vanhommerig, Maarten F. Schim van der Loeff, Ronald B. Geskus, Henry J. C. de Vries, Jan M. Prins, Maria Prins, Sylvia M. Bruisten

Figure 1 is incorrect. The correct Figure 1 can be viewed here:

**Figure:**